# Quality of Life and Experiences of Patients with Gastrointestinal Stromal Tumors (GIST) on Imatinib Treatment

**DOI:** 10.1007/s12029-026-01417-x

**Published:** 2026-03-10

**Authors:** Kim Westerdijk, Neeltje Steeghs, Winette T. A. van der Graaf, Joost S. Groen, Nielka P. van Erp, Rosella P. M. G. Hermens, Ingrid M. E. Desar

**Affiliations:** 1 Department of Medical Oncology, Radboud University Medical Center, Radboud Institute for Medical Innovation, Nijmegen, the Netherlands; 2 Department of Medical Oncology, Netherlands Cancer Institute, Antoni Van Leeuwenhoek, Amsterdam, the Netherlands; 3 Department of Medical Oncology, Erasmus MC Cancer Institute, Rotterdam, the Netherlands; 4 Patient Advocacy Group “Patiëntenplatform Sarcomen”, Utrecht, the Netherlands; 5 Department of Pharmacy, Radboud Institute for Medical Innovation, Radboud University Medical Center, Nijmegen, The Netherlands; 6 Department of IQ Health, Radboud University Medical Center, Nijmegen, the Netherlands

**Keywords:** Imatinib, Gastrointestinal stromal tumour, Quality of Life, Patients' experiences

## Abstract

**Purpose:**

Adjuvant or palliative treatment with imatinib improved the survival of patients with rare gastrointestinal stromal tumours (GISTs) impressively. However, the impact on quality of life (QoL) and patients’ experiences with imatinib is largely unknown. We performed a survey study in order to assess QoL and experiences with imatinib treatment, comparing the adjuvant and metastatic setting.

**Methods:**

Patients with GIST who were on active imatinib completed a cross-sectional web-based survey with the following questionnaires: EORTC Quality of Life Questionnaire (QLQ-C30), Treatment Satisfaction Questionnaire for Medication (TSQM), Beliefs about Medicines Questionnaire (BMQ), Cancer Worry Scale (CWS) and Medication Adherence Report Scale (MARS).

**Results:**

Symptom burden and the scores for the QLQ-C30 scales were similar between adjuvant (*n* = 19) and metastatic disease (*n* = 56), with a mean(SD) global QoL score of 69.2 in the entire study population (*n* = 77). Patients with metastatic disease experienced less side effects (63.0(20.3) versus 51.0(22.7); *p* = 0.035), had better global satisfaction scores (79(15.9) versus 68(18.8); *p* = 0.015) and are more convinced of the necessity of imatinib for controlling the disease compared to patients receiving adjuvant treatment (score 19.9(4.6) versus 17.5(3.6); *p* = 0.043). Approximately 3 out of 4 patients report high fear of cancer recurrence / progression, without a difference in the adjuvant or metastatic setting. Therapy adherence was high (96.1%).

**Conclusion:**

Patients with GIST on imatinib treatment have good QoL but show high percentage of fear for cancer recurrence / progression. Especially patients with metastatic disease believe in the necessity of imatinib. These findings help to educate and support individual patients treated with imatinib.

## Introduction

Gastrointestinal stromal tumours (GISTs) are mesenchymal tumours that can arise in any part of the gastrointestinal (GI) tract, but occur most frequently in the stomach or small intestine [1]. They have a reported incidence ranging from 7 to 15 cases per million per year [2, 3].

GISTs have shown to respond poorly to conventional chemotherapy [4, 5]. However, after it was reported that activation of the cytokine receptor (c-KIT) is critical in the pathogenesis of GIST, imatinib was approved as first-line treatment for GIST in 2001 and the prognosis of patients with GIST improved dramatically [6–8]. Imatinib is a tyrosine kinase inhibitor (TKI) targeting the KIT-receptor [9, 10]. This oral drug is registered for both adjuvant treatment and for metastatic disease [11, 12]. In the adjuvant setting, imatinib is prescribed for three years. The median time for patients with metastatic GIST on imatinib therapy is 68 months [13].

Information on clinical outcomes of imatinib treatment is extensive, though studies on quality of life (QoL) and patients’ experiences with their treatment with imatinib are limited [14–17]. This was addressed as an unmet need by the (inter)national patient advocacy groups [18]. Therefore, we performed a survey study among patients with GIST who are treated with imatinib in order to assess their QoL and experiences with treatment, comparing the adjuvant and metastatic setting.

## Methods

### Study Design and Population

We performed a cross-sectional web-based survey amongst patients with GIST on active treatment with imatinib in collaboration with the Dutch GIST Patient Advocacy Group. Exclusion criterion was a lack of understanding of the Dutch language. This study was performed in line with the Dutch Code of Conduct for Research Integrity. The study protocol was considered not to be subject to the Medical Research Involving Human Subjects Act by the local Medical Ethics Committee of the Radboudumc in Nijmegen, the Netherlands (file number 2022–16004).

### Study Measures

QoL of GIST patients treated with imatinib was investigated using the Quality of Life Questionnaire (QLQ-C30) and 16 symptom-related questions from the EORTC Item Library. To assess experiences of patients on imatinib treatment we used the Treatment Satisfaction Questionnaire for Medication (TSQM), the Beliefs about Medicines Questionnaire (BMQ) and the Cancer Worry Scale (CWS). In order to describe the study population, we collected information regarding basic patient demographics such as age, gender and baseline tumor- and treatment characteristics. We also determined self-reported medication adherence using the Medication Adherence Report Scale (MARS), since medication adherence affects the outcome measures.

The QLQ-C30 investigates health-related QoL (HRQoL) and includes both multi-item scales and single-item measures [19]. For the symptom scales and single items, a higher score indicates more burden. For the other scales, a higher score indicates healthier level of functioning. For the QLQ-C30 summary score a higher score indicated a better HRQoL [20]. Clinically important differences were determined according to the guidelines of the EORTC [21].

To investigate symptom burden of imatinib treatment, 16 symptom-related questions from the EORTC Item Library were selected. This selection was based on the most frequently reported side effects in (registration) studies for imatinib and the top 10 of patient-reported symptoms [7, 19, 22, 23]. For these symptom-related questions a higher score also indicates more burden.

Satisfaction with imatinib treatment was investigated with the TSQM version 1.4 [24]. It consists of questions on four domains (Effectiveness, Convenience, Side Effects and Global Satisfaction) [24]. Higher scores indicate better satisfaction.

The BMQ comprises of two parts (specific and general) [25]. The BMQ-specific investigates patients’ beliefs regarding the necessity of their prescribed medication for controlling the disease and concerns regarding adverse effects. The BMQ-general investigates more general beliefs that medicines are harmful and overused by physicians. Higher scores indicate higher beliefs. A necessity-concerns differential represents the cost–benefit analysis for each patient. The harm-overuse differential represents the balance between confidence in the prescribing physician and beliefs in harmfulness of medication in general [25, 26].

The CWS evaluates patients fear of cancer recurrence or progression, where higher scores indicate higher worries. In this study the six item version of the CWS was used, where a score ≥ 10 and ≥ 12 indicate high and severe fear of recurrence/progression, respectively [27].

The MARS contains 5 statements regarding therapy-adherence, where a total score ≥ 21 or a score of 4 on each individual item represents therapy-adherence [28].

### Data Collection

The questionnaires were converted into one web-based questionnaire using Limesurvey (https://manual.limesurvey.org).

Patients were invited to fill in the questionnaire by an email sent by the Dutch GIST Patient Advocacy Group to all its members (estimated approximately 150–200 patients on imatinib). In the email, patients who were on active treatment with imatinib were requested to fill in the questionnaire. A patient information sheet was attached to the email, explaining the goal of the study. Patients could open the questionnaire by activating the hyperlink attached to the email. In order to start the actual questionnaire, electronic informed consent was required. The first part of the questionnaire consisted of 12 questions about baseline characteristics and treatment with imatinib. The second part comprised of the validated questionnaires as described above. A reminder to the questionnaire was sent once and completion of the total questionnaire took approximately 15 min. Data was collected in December 2022.

### Data Analysis

The questionnaire data were collected in Limesurvey and exported into IBM SPSS statistics for Windows, version 27.0 (IBM Corp., Armonk, NY, USA). The questionnaires were included when at least 50% of the questions were completed. Descriptive statistics was used to describe characteristics of the patients and their treatment. Scorings of the validated questionnaires were calculated as described in the respective scoring manuals. Independent samples t-test was used to identify differences in the questionnaire scores for treatment setting (adjuvant versus metastatic). Association between baseline characteristics and questionnaire outcomes were tested by univariate analysis using a t-test or ANOVA where appropriate. Variables that showed statistical significance in the univariate analysis were subsequently tested in a multivariate analysis.

## Results

The survey yielded a total of 85 responses, of which 75 (88.2%) were completed for at least 50% and included in the analysis. Patient characteristics are shown in Table 1. Patients had a median (range) age of 67 (34–90) years. Patients on adjuvant treatment (*n* = 19) received imatinib for a median (range) duration of 22 (2–36) months versus 72 (1–260) months for patients with metastatic disease (*n* = 56) (*p* =  < 0.001). Scorings for all questionnaires are reported in Table 2. Univariate analysis did not identify any relationship between patient characteristics and the questionnaire outcomes.

**Table 1:** Baseline characteristics

*Patients (n)*	Adjuvant setting(*n* = 19)	Metastatic setting(*n* = 56)	Total (*n* = 75)
Age (median (range)) in years	63 (34–77)*	68 (44–90)*	67 (34 – 90)
Gender (*n* (%))			
Male	9 (47.4)	24 (43.6)	33 (42.9)
Female	10 (52.6)	31 (56.4)	43 (55.8)
Unknown	-		1 (1.3)
Country of birth (*n* (%))			
Netherlands	18 (94.7)	47 (83.9)	67 (87)
Other	1 (5.3)	9 (16.1)	10 (13)
Educational level (*n* (%))			
Low^a^	3 (15.8)	17 (30.4)	20 (26.0)
Intermediate^b^	5 (26.3)	13 (23.2)	18 (23.4)
High^c^	11 (57.9)	26 (46.4)	39 (50.6)
Occupational status (*n* (%))			
Unemployed	0 (0.0)	3 (5.4)	3 (3.9)
Employed	8 (42.1)*	9 (16.1)*	18 (23.4)
Incapacitated	2 (10.5)	9 (16.1)	11 (14.3)
Retired	9 (47.4)	35 (62.5)	45 (58.4)
Household (*n* (%))			
Partner	10 (52.6)	42 (75.0)	53 (68.9)
Partner and children	5 (26.3)*	3 (5.4)*	9 (11.7)
Children	0 (0.0)	1 (1.8)	1 (1.3)
One-person	4 (21.1)	9 (16.1)	13 (16.9)
Unknown	0 (0.0)	1 (1.8)	1 (1.3)
Years since diagnosis (median (range))	2 (0 – 9)*	7 (0 – 22)*	6 (0 – 22)
Treatment setting (*n* (%))			
Neo-adjuvant	-	-	2 (2.6)
Adjuvant	19	-	19 (24.7)
Metastatic	-	56	56 (72.7)
Duration of treatment with imatinib in months (median (range))	22 (2 – 36)*	72 (1 – 260)*	35 (1 – 260)
Dose adjustment during treatment with imatinib (*n* (%))			
Yes	13 (68.4)	27 (48.2)	36 (46.8)
No	6 (31.6)	29 (51.8)	41 (53.2)

^a^low educational level: primary education, lower general secondary education, preparatory secondary vocational education

^b^intermediate educational level: secondary vocational education, higher general secondary education, pre-university education

^c^high educational level: higher vocational education, academic education

^*^ statistically significant difference

**Table 2:** Results questionnaires

Questionnaires	Score (mean (SD) or number (%))		
	Adjuvant setting (*n* = 19)	Metastatic setting (*n* = 56)	Total (*n* = 77)
**EORTC QLQ-C30**			
Functional scales			
Physical functioning	82.5 (16.4)	81.4 (19.5)	82.1 (18.6)
Role functioning	59.3 (29.8)	73.9 (27.0)	70.9 (28.1)
Emotional functioning	77.2 (27.1)	80.3 (17.6)	79.5 (20.1)
Cognitive functioning	73.7 (33.0)	80.0 (22.8)	78.9 (25.6)
Social functioning	63.2 (32.2)*	80.9 (20.9)*	76.8 (25.1)
Symptom scales			
Fatigue	41.8 (25.1)	32.1 (23.8)	33.8 (24.5)
Nausea and vomiting	12.3 (12.2)	12.4 (21.1)	12.3 (18.9)
Pain	5.3 (11.2)	13.0 (21.7)	11.2 (19.7)
Dyspnoea	21.1 (29.8)	14.5 (23.8)	15.8 (25.2)
Insomnia	22.8 (31.5)	26.7 (30.4)	25.4 (30.2)
Appetite loss	15.8 (20.4)	13.9 (24.6)	14.0 (23.3)
Constipation	5.3 (16.7)	6.7 (17.5)	6.1 (17.0)
Diarrhoea	38.6 (35.6)	27.3 (32.1)	29.4 (33.1)
Financial difficulties	9.3 (15.4)	7.3 (18.9)	8.0 (18.0)
Global health status / QoL	65.8 (20.6)	69.8 (17.5)	69.2 (18.2)
Summary score	75.6 (17.4)	80.8 (13.2)	79.9 (14.4)
**Single items EORTC Item Library**			
Swelling in any part of the body	19.3 (25.6)	15.2 (24.7)	15.8 (24.6)
Swelling of the face or around the eyes	47.4 (33.9)	37.6 (30.8)	39.9 (31.7)
Flatulence	40.4 (30.6)	34.5 (29.4)	36.0 (29.2)
Indigestion or heartburn	26.3 (32.5)	17.0 (23.0)	18.9 (25.7)
Abdominal pain or cramps	28.1 (25.5)	19.1 (23.0)	21.3 (23.7)
Hair loss	16.7 (28.6)	20.0 (25.3)	19.1 (25.8)
Food and drink tasting different from usual	26.3 (32.5)	15.2 (23.8)	18.4 (26.3)
*Muscle aches, pains, or cramps*	40.4 (30.6)	48.8 (35.3)	45.3 (34.5)
Aches or pains in joints	19.3 (27.9)	28.5 (31.0)	25.4 (30.2)
Headache	17.5 (23.2)	11.5 (20.5)	12.7 (21.1)
Chest pain	5.3 (12.5)	3.6 (10.5)	3.9 (10.8)
Itchy skin	12.3 (19.9)	16.0 (25.7)	15.6 (24.7)
Skin rash	12.3 (16.5)	8.5 (17.2)	10.1 (18.1)
Dry skin	14.0 (20.2)	15.4 (23.1)	14.7 (22.1)
Easy bruising	14.0 (23.1)	26.7 (33.6)	23.2 (31.3)
**BMQ-specific**			
Necessity	17.5 (3.6)*	19.9 (4.6)*	19.2 (4.4)
Concerns	13.7 (4.2)	13.2 (3.9)	13.4 (4.0)
Necessity-concerns differential	3.7 (3.7)*	6.6 (5.1)*	5.9 (5.0)
**BMQ-general**			
Harm	8.6 (3.2)	9.1 (2.6)	8.9 (2.8)
Overuse	9.4 (3.5)	10.6 (2.9)	10.3 (3.1)
Harm-overuse differential	-0.7 (3.1)	-1.5 (2.5)	-1.3 (2.7)
**MARS**			
Adherence	18 (94.7)	53 (94.6)	73 (94.8)
Nonadherence	1 (5.3)	2 (3.6)^a^	3 (3.9)
**TSQM questionnaire**			
Domain global satisfaction	68.0 (18.8)*	79.0 (15.9)*	76.5 (17.2)
Domain effectiveness	68.3 (13.3)	71.3 (19.0)	70.5 (17.6)
Domain side effects	51.0 (22.7)*	63.0 (20.3)*	60.4 (21.6)
Domain convenience	77.2 (15.4)	79.4 (16.4)	78.9 (16.1)
**CWS**			
CWS total score			12.2 (3.5)
High fear of recurrence (score ≥ 10)	13 (68.4)	41 (74.5)	56 (73.7)
Severe fear of recurrence (score ≥ 12)	9 (47.4)	30 (54.5)	40 (52.6)

Abbreviations: *BMQ* Beliefs about Medicines Questionnaire; *CWS* Cancer Worry Scale; *EORTC* European Organisation for Research and Treatment of Cancer; *MARS* Medication Adherence Report Scale; *QLQ-C30* Quality of Life Questionnaire; *QoL* Quality of Life; *SD* standard deviation; *TSQM* Treatment Satisfaction Questionnaire for Medication;

^*^ statistically significant difference

^a^ data from one patient is missing

### Quality of Life and Symptom Burden

**Fig. 1:**
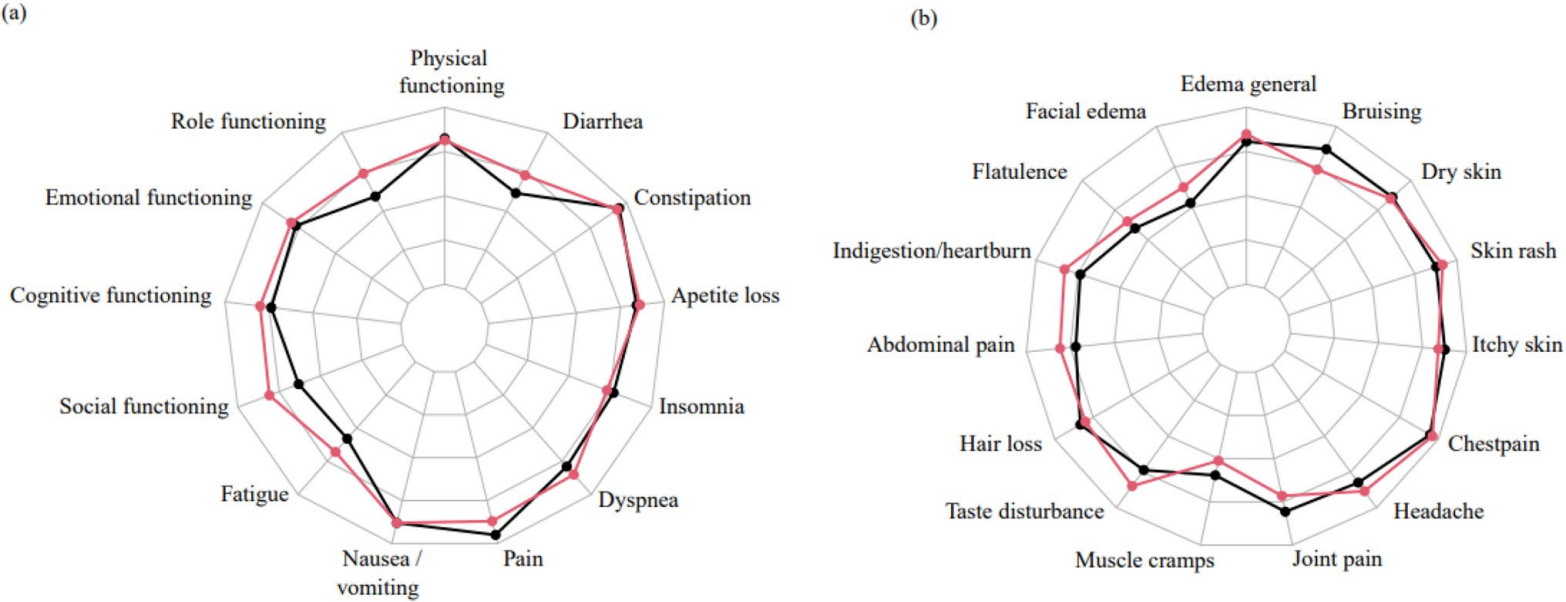
Quality of life and symptom burden. Spider plot of the EORTC QLQ-C30 scale scores (**a**) and symptom items from the EORTC Item Library (**b**). The plot is showing scores of the patients receiving adjuvant treatment (black line) or treatment for metastatic disease (red line). Scores of symptom scales and single items were reversed to obtain uniform direction of all scales. Scores range from 0 to 100, with higher scores indicating better functioning or less symptoms

The mean (SD) global QoL score and summary score for the QLQ-C30 in the entire study population was 69.2 (18.2) and 79.9 (14.4) points, respectively. Most burdensome side effects impacting quality of life seem to be fatigue, facial edema and muscle cramps. Overall, the scores for the QLQ-C30 scales and EORTC single items were similar between patients receiving imatinib in the adjuvant and metastatic setting (Fig. 1). However, patients with metastatic disease had a significantly higher (better) score (mean (SD)) for social functioning compared to patients receiving adjuvant treatment (80.9 (20.9) versus 63.2 (32.2), *p* = 0.007), which is considered a large clinical difference [21]. There were no significant differences in symptom burden between the two groups (Table 2).

### Treatment Satisfaction

For all patients, the score on the side effects domain was significantly lower (worse) compared to the other domains (*p* < 0.001). Patients with metastatic disease scored significantly higher (better) on the side effects domain compared to patients receiving adjuvant treatment (63.0 (20.3) versus 51.0 (22.7); *p* = 0.035). Furthermore, their score for global satisfaction was higher (79 (15.9) versus 68 (18.8); *p* = 0.015).

### Beliefs about Medicines

Patients with metastatic disease are more convinced of the necessity of imatinib for controlling the disease compared to patients receiving adjuvant treatment (score 19.9 (4.6) versus 17.5 (3.6); *p* = 0.043). Furthermore, patients with metastatic disease are more convinced that the positive effects of imatinib outweigh concerns regarding potential negative effects of imatinib compared to patients receiving adjuvant treatment (necessity-concerns differential 6.6 (5.1) versus 3.7 (3.7); *p* = 0.028).

### Fear of Cancer Recurrence / Progressive Disease

A total of 56 patients (73.7%) and 40 patients (52.6%) had high or severe fear of cancer recurrence or cancer progression, respectively. There were no significant differences between patients in the adjuvant or metastatic setting.

### Medication Adherence

A total of 96.1% of the entire study population was considered to be therapy-adherent based on the MARS.

## Discussion

In this cross-sectional study we investigated QoL and experiences with imatinib treatment in patients with GIST and explored differences between patients treated in the adjuvant and metastatic setting. Overall, self-reported therapy adherence was high and results from the questionnaires were comparable between both groups. However, patients with metastatic disease scored better on global satisfaction, social functioning and reported fewer side effects according to TSQM 1.4.

Although imatinib has been registered for treatment of GIST for over 20 years, studies reporting on QoL and experience with imatinib treatment in GIST patients are limited and have mostly included patients without active imatinib treatment or included patients with a broad variety of cancer types [14–17, 29]. The QLQ-C30 scores in our study were comparable to previous reports of patients receiving oral anticancer treatment [14, 15, 30]. The score for social functioning was higher in patients with metastatic disease, which is in contrast with a previous study [31]. Perhaps this is related to the relatively long duration of treatment of the GIST patients treated in the metastatic setting (72 months) in our study in respect to other cancer types. This long duration suggests these patients respond well to treatment and experience limited side effects, possibly explaining their high score on social functioning.

Overall, this study demonstrated that patients with GIST and especially patients with metastatic disease were convinced of the positive effect of imatinib. This has not been described in literature and seems in contrast with the curative goal of adjuvant treatment. However, one might hypothesize that patients receiving palliative treatment are more aware of the life-threatening disease and when having tumour-related symptoms they may benefit from positive effects of the medication, whereas with adjuvant treatment it is unsure which patients benefit from treatment with imatinib. Furthermore, the longer median time on treatment suggests adaption and selection bias since patients with severe adverse events might have stopped earlier. This might also explain the higher score on the side effects domain in the metastatic compared to adjuvant setting. The potential benefits of adjuvant treatment are huge though, since it has been shown that imatinib significantly increases recurrence-free survival compared to placebo [32]. Therefore, knowledge of these beliefs of patients can help physicians in their education of patients.

A total of 56 patients (73.7%) and 40 patients (52.6%) in this study had high or severe fear of cancer recurrence / progression, respectively, without a difference in the adjuvant or metastatic setting. These percentages are comparable to a previous study in GIST patients [33]. A study by van de Wal et al. reported lower fear of cancer recurrence / progression, however they also included patients who were not on active imatinib treatment [29]. It was also demonstrated that QoL is significantly affected by fear of cancer recurrence / progression [34]. Therefore, fear should be discussed, monitored and support should be provided in all GIST patients treated with imatinib, regardless of the treatment setting.

This study was designed in a truly patient included way; the topic of interest, the selection of relevant questionnaires, the invitation to participants as well as the interpretation of the data and writing of the manuscript was in close collaboration. However, this also results in some limitations. The patients included were invited by an email to all members of the GIST Patient Advocacy Group. For a rare cancer as GIST, the sample size of 75 is adequate but we were not able to calculate an exact response rate due to privacy regulations of the Patient Advocacy Group. Based on the by the Patient Advocacy Group estimated numbers, between 150–200 of their members are considered on active imatinib treatment. This results in an estimated response rate between 37 and 50 percent. Selection bias is possible, since 1) patients not member of the Patient Advocacy Group were not invited, 2) patients responding to questionnaires are in general more motivated, older, higher educated and more satisfied [35, 36] and 3) only ongoing patients were invited. This could have limited the generalizability of our findings. However, a previous study reported significant differences in the reporting of side effects amongst patients on current versus former TKI treatment [16]. For antidepressants it has been described more extensively that patients’ perspective on side effects changes after discontinuation of a drug [37]. Due to the cross-sectional study design, the median treatment duration of patients included is relatively long. Patients who discontinued imatinib treatment due to disease progression or toxicity are therefore underrepresented. Furthermore, the cross-sectional design limits the ability to investigate causality between treatment course, treatment setting and QoL and treatment experiences. Currently, a longitudinal study is in progress investigating long-term survivorship challenges of advanced/metastatic GIST patients responding to imatinib treatment (EORTC-1944-QLG-STBSG). However, despite its limitations, this study provides valuable insight in QoL and experiences with imatinib treatment in GIST patients. Physicians could ask patients for their beliefs regarding the beneficial effect of their medication and provide more individual guidance in underlining the necessity of their medication, especially in patients in the adjuvant setting. Furthermore, considering the high percentage of fear of recurrence / progression, this topic could receive more attention in patient visits, since this significantly affects QoL. Future research should focus on identifying specific patient characteristics related to worse QoL or a more negative treatment experience in order to identify patients at risk and on successful interventions addressing this fear.

In this study we showed that patients with GIST who are treated with imatinib have good HRQoL, report to be adherent to therapy and show high percentage of fear for cancer recurrence/progression. Furthermore, patients believe in the necessity of imatinib, although this belief is more pronounced in patients with metastatic disease who also reported fewer side effects. These findings help to select appropriate education measures and support for individual patients in both the adjuvant and metastatic setting.

## Data Availability

The data underlying this article will be shared on reasonable request to the corresponding author.
